# Synthesis and Use of the Bifunctional Sulfenic Acid Probe BCN‐E‐BCN for *In Vitro* and Cell‐Based Assays of Protein Oxidation

**DOI:** 10.1002/cpz1.559

**Published:** 2022-10-06

**Authors:** Katarina Micovic, Thershan Satkunarajah, Alexandre Carnet, Mackenzie Hurst, Russell Viirre, Michael F. Olson

**Affiliations:** ^1^ Department of Chemistry and Biology Toronto Metropolitan University Toronto Ontario Canada

**Keywords:** click chemistry, protein oxidation, sulfenic acid

## Abstract

The reversible oxidation of cysteine thiol groups to sulfenic acid by reactive oxygen species (ROS) such as hydrogen peroxide can impact protein function, activity, and localization. As a consequence, ROS have profound effects on cell functions including proliferation, differentiation, and survival. Furthermore, there are clear associations between the effects of ROS on cells and the etiology of several diseases including cancer and neurodegeneration. In spite of the importance of cysteine sulfenylation as a validated post‐translational modification, its labile nature impedes efficient and reproducible detection of proteins with cysteine sulfenic acid residues. To overcome this challenge, we developed a novel cell‐permeable bifunctional reagent, consisting of two linked bicyclo[6.1.0]nonyne (BCN) moieties coupled with a short ethylenediamine‐derived linker (BCN‐E‐BCN) that enables the detection of sulfenylated proteins *in vitro* and in intact cells. The two symmetrical BCN groups allow protein sulfenic acids to be selectively tagged with a BCN at one end while allowing for copper‐free click chemistry with azide‐tagged reagents of the opposite BCN. In this protocol, the synthesis of BCN‐E‐BCN and its use to detect cysteine sulfenic acids will be detailed. © 2022 The Authors. Current Protocols published by Wiley Periodicals LLC.

**Basic Protocol 1**: Copper‐mediated cyclopropanation of 1,5‐cyclooctadiene

**Basic Protocol 2**: Synthesis of *endo‐* and *exo‐*bicyclononyne

**Basic Protocol 3**: Synthesis of *endo‐*BCN‐E‐BCN

**Basic Protocol 4**: BCN‐E‐BCN treatment of wild‐type and cysteine‐deficient mutant recombinant cofilin protein

**Basic Protocol 5**: BCN‐E‐BCN labeling in live cells

**Basic Protocol 6**: Western blotting and visualization of BCN‐E‐BCN‐labeled samples

## INTRODUCTION

**Figure 1:**
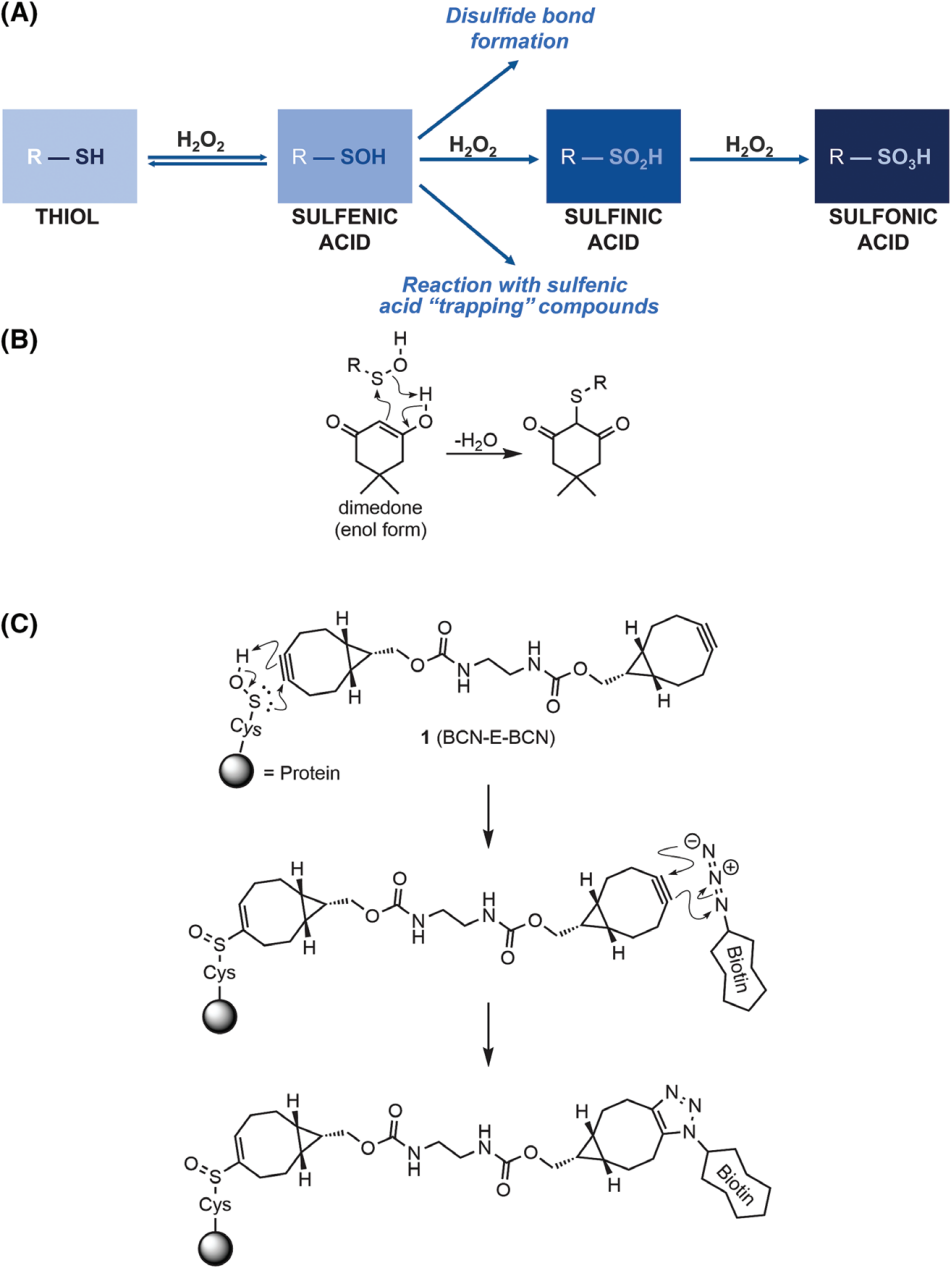
Stages of cysteine thiol oxidation by H_2_O_2_ and covalent labeling of sulfenic acids: (**A**) When exposed to H_2_O_2_, cysteine thiols are oxidized to form sulfenic acid. From there, it can be reduced, form disulfide bonds, or become further irreversibly oxidized. The sulfenic acid stage is highly reactive, allowing for irreversible labeling with compounds such as dimedone or BCN‐E‐BCN. (**B**) Nucleophilic addition of the enol form of dimedone onto the electrophilic sulfur atom of sulfenic acid with the ejection of a water molecule results in the dimedone thioether; (**C**) A BCN group from **1** undergoes a pericyclic ene–type reaction with sulfenic acid, resulting in an alkenyl sulfoxide linkage. Subsequently, an azide‐containing reporter molecule (e.g., azide‐PEG3‐biotin) is added and this undergoes a copper‐free, strain‐promoted azide‐alkyne cycloaddition reaction, forming a 1,2,3‐triazole linkage between the protein‐probe complex and reporter.

Proteins undergo a variety of post‐translational modifications (PTMs) that may result in alterations to properties including their function and activity, stability, interactions with proteins or ligands, or subcellular localization. Well‐established PTMs, such as phosphorylation, ubiquitylation, and prenylation, involve the covalent attachment of a molecule to specific recipient amino acid residues. Although many of these PTMs are very well characterized, there are additional PTMs that are less widely studied, often due to a paucity of convenient research tools. One example is the oxidation of cysteine thiols by reactive oxygen species (ROS) such as hydrogen peroxide (H_2_O_2_), which acts as an important second messenger in cells for redox signaling (Rhee, 1999). Once cysteine residues are oxidized to sulfenic acid, they can react with other cysteine sulfenic acids to form disulfide bridges or be further irreversibly oxidized to sulfinic and sulfonic acid states (Fig. 1A). The importance of cysteine thiol modifications has been highlighted in many situations, including the response of the metabolic system to redox stress (van der Reest, Lilla, Zheng, Zanivan, & Gottlieb, 2018), or central roles of protein oxidation in several diseases (Fra, Yoboue, & Sitia, 2017), including type 2A multiple endocrine neoplasia (MEN2A) (Qiao et al., 2014). The challenge to increase our knowledge of cysteine oxidation in health and disease is the development of reliable methods to characterize and identify oxidized cysteine thiols.

In order to study the role of cysteine sulfenic acids in physiological pathways and processes, several methods to “trap” them in this state have been developed. The cyclic diketone dimedone and its derivatives have been widely used as cysteine sulfenic acid probes in various applications (Forman et al., 2017; Nelson et al., 2010; Paulsen & Carroll, 2009; Qian et al., 2012). As depicted in Figure 1B, the enol form of dimedone acts as a nucleophile toward the electrophilic sulfur atom of sulfenic acids, forming a covalent thioether linkage (Benitez & Allison, 1974). However, the utility of dimedone‐based probes is limited because of their slow reaction rates with sulfenic acids (Heppner et al., 2018). As a result, dimedone requires longer incubation times, which increases the possibility that a significant proportion of sulfenic acid residues escape detection through reduction to thiols, the formation of disulfide bonds, or further irreversible oxidation. To overcome these limitations, a bifunctional reagent **1**, consisting of two bicyclo[6.1.0]nonyne moieties (BCN) coupled *via* an ethylenediamine‐derived linker (BCN‐E‐BCN) was developed as an alternative sulfenic acid probe (Fig. 1C) (McGarry, Shchepinova, Lilla, Hartley, & Olson, 2016). As depicted in Figure 1C, the BCN group undergoes a rapid pericyclic ene–type reaction with sulfenic acid, forming cyclooctenyl sulfoxide with a reaction rate constant hundreds of times faster than that of the dimedone reaction (Poole et al., 2014). The novel probe **1** can detect oxidized cysteine residues in proteins with great effectiveness by first undergoing this reaction with one end of the symmetrical bifunctional probe. Subsequently, it exposes the remaining BCN group to an azide‐based reagent to undergo another rapid and efficient copper‐free strain‐promoted azide‐alkyne cycloaddition, enabling detection or enrichment. For example, reaction of azide‐PEG3‐biotin with a free BCN allows for visualization of oxidized proteins by western blotting using fluorescent dye–labeled streptavidin to detect biotinylated protein samples. Biotinylation would also allow for enrichment of oxidized proteins by chromatography with streptavidin‐conjugated beads, which could then be subjected to mass spectrometry analysis to identify the enriched proteins. In addition, azide‐conjugated fluorophores would also enable the visualization of BCN‐E‐BCN labeling of oxidized proteins in fixed cells, allowing for the subcellular localization of this PTM to be examined. Several studies have used BCN‐E‐BCN, such as those on the oxidation of Src kinases and its role in redox signaling (Camargo et al., 2022; Heppner et al., 2018), and examination of how protein oxidation affects endoplasmic reticulum (ER) stress responses (Camargo et al., 2018), demonstrating the utility of BCN‐E‐BCN for exploring the oxidation of proteins in a variety of contexts.

Basic Protocols 1 to 3 describe the steps involved in synthesizing BCN‐E‐BCN. Once BCN‐E‐BCN is produced, Basic Protocol 4 outlines the *in vitro* oxidation of a recombinant protein, using purified recombinant cofilin incubated with hydrogen peroxide as an exemplar, followed by labeling with azide‐PEG3‐biotin *via* copper‐free click chemistry. Support Protocol 1 covers the purification of cofilin from *E. coli*. Basic Protocol 5 outlines a procedure for labeling live cells with BCN‐E‐BCN and reacting cell lysates with azide‐PEG3‐biotin. Finally, Basic Protocol 6 describes the western blotting protocol for visualizing oxidized cysteine residues labeled with BCN‐E‐BCN following biotinylation.

## Basic Protocol 1 COPPER‐MEDIATED CYCLOPROPANATION OF 1,5‐CYCLOOCTADIENE 2

**Figure 2:**
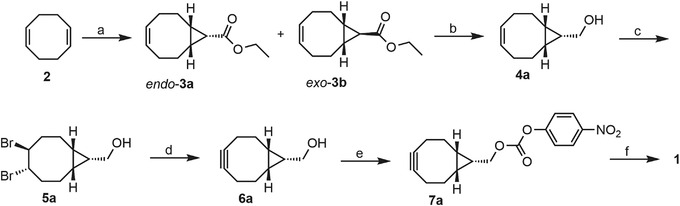
BCN‐E‐BCN synthesis. Reagents and conditions: (**A**) CuSO_4_, ethyl diazoacetate, n‐hexane, 68°C, 2 hr, *endo*‐3a: 14%, *exo*‐3b: 28%; (**B**) LiAlH_4_, THF, 0°C to rt, 1 hr, 92%; (**C**) Br_2_, DCM, 0°C to rt, 1 hr, 92%; (**D**) KO*t*Bu, THF, 0 to 66°C, 2 hr, 42%; (**E**) *p*‐nitrophenyl chloroformate, pyridine, DCM, rt, 1 hr, 78%; (**F**) ethylenediamine, NEt_3_, DMF, rt, 2 hr, 58%. Cyclooctadiene was subjected to cyclopropanation using inexpensive copper sulfate as the catalyst (Ghandi & Mashayekhi, 2007). The isomers were separated by column chromatography and then reduced under standard conditions with LiAlH_4_ (Dommerholt et al., 2010). When quenching with distilled water, drying over magnesium sulfate and subsequent filtration resulted in a gel; dilute HCl was used in place of distilled water, followed by aqueous workup. Bromination affords the respective dibromide (Li et al., 2017) as white needles after recrystallization in heptane. In our experience with carrying over a yellow‐brownish oil to the elimination step, the reaction yields have been poor. Moreover, emulsions were commonly encountered during extraction, which warrants rotary evaporation of the reaction solvent after quenching any excess potassium *tert*‐butoxide with ammonium chloride. Column chromatography using a mixture of ethyl acetate in dichloromethane affords the strained alkyne as a white solid (Dommerholt et al., 2010; Li et al., 2017). Coupling with *p*‐nitrophenyl chloroformate followed by ethylenediamine affords sulfenic acid probe **1** in good yields (McGarry et al., 2016).

This protocol (Fig. 2) describes the preparation of *endo*‐ and *exo*‐ethyl bicyclo[6.1.0]non‐4‐ene‐9‐carboxylate **3a** and **3b** using commercially available diene **2**, ethyl diazoacetate solution, and anhydrous copper (II) sulfate. The compound was prepared with modifications to a previously published procedure (Ghandi & Mashayekhi, 2007). The preferred stereoisomer *endo*‐**3a** was purified by normal phase column chromatography and carried through the rest of the synthesis as a single isomer. Throughout this paper, the notation “**b**” in compound numbers refers to the *exo*‐stereoisomer, even though the compound is not depicted in Figure 2. NMR spectra for the synthesized compounds are provided in the Supporting Information section.

### Materials

1,5‐Cyclooctadiene (Sigma Aldrich)
Hexane (ACP Chemicals)
Anhydrous copper (II) sulfate
Ethyl diazoacetate (contains 10 wt.% DCM, Fluka)
SiliaFlash P60 silica gel (40‐63 µM, SiliCycle)

2‐neck round bottom flask, 250 ml
Magnetic stir bar
Pressure‐equalizing addition funnel
Rotary evaporator
Chromatography column
NMR spectrometer

### Cyclopropanation of 2

1 Charge a flame‐dried, 2‐neck 250‐ml round bottom flask with a magnetic stir bar, 1,5‐cyclooctadiene **2** (8.0 ml, 65.2 mmol), hexane (180 ml), and anhydrous copper (II) sulfate (2.600 g, 10.2 mmol).
2 Heat the suspension to reflux (>68°C) with moderate stirring.
3 While the mixture is at reflux, using a pressure‐equalizing addition funnel, add a solution of ethyl diazoacetate (7.6 ml, 64.7 mmol) diluted in hexane (17 ml) dropwise over 30 min.
4 Stir the reaction mixture at reflux for an additional 2 hr.
5 Filter the hot suspension by gravity filtration and concentrate the resulting brown solution using a rotary evaporator.
6 Purify the material by column chromatography, eluting with 0:1 → 1:4 dichloromethane in hexane.
7 Yield 42% (1:2 *endo‐* **3a**:*exo‐* **3b**). TLC (1:3 dichloromethane in heptane): R_f_ = 0.31 (*endo‐* **3a**), 0.24 (*exo‐* **3b**); *endo‐* **3a**: ^1^H NMR (CDCl_3_, 400 MHz): δ 5.65‐5.57 (m, 2H), 4.12 (q, *J* = 7.1 Hz, 2H), 2.56‐2.45 (m, 2H), 2.26‐2.14 (m, 2H), 2.10‐2.00 (m, 2H), 1.87‐1.77 (m, 2H), 1.70 (t, *J* = 8.8 Hz, 1H), 1.44‐1.33 (m, 2H), and 1.26 (t, *J* = 7.1 Hz, 3H) ppm. ^13^C NMR (CDCl_3_, 100 MHz): δ 172.3, 129.5, 59.8, 27.1, 24.2, 22.7, 21.3, and 14.5 ppm. *exo‐* **3b**: ^1^H NMR (CDCl_3_, 400 MHz): δ 5.66‐5.59 (m, 2H), 4.10 (q, *J* = 7.1 Hz, 2H), 2.32‐2.25 (m, 2H), 2.22‐2.15 (m, 2H), 2.11‐2.03 (m, 2H), 1.60‐1.42 (m, 4H), 1.24 (t, *J* = 7.1 Hz, 3H), and 1.17 (t, *J* = 4.6 Hz, 1H) ppm. ^13^C NMR (CDCl_3_, 100 MHz): δ 174.6, 130.0, 60.4, 28.4, 28.0, 27.8, 26.8, and 14.4 ppm. The spectroscopic data are consistent with those reported in the literature (Dommerholt et al., 2010).
8 Dried and purified **3a** and **3b** can be stored indefinitely if sealed under nitrogen or argon, stored cold (4°C), and protected from light (Table 1).

**Table 1:** Troubleshooting Guide for the Cyclopropanation of 1,5‐Cyclooctadiene

Problem	Possible cause	Solution
Poor yield	Copper catalyst has moisture	Dry the copper (II) sulfate beforehand and ensure a light grey solid is used
	Rate of addition of ethyl diazoacetate is too fast	Add ethyl diazoacetate at a consistent rate over 30 min
	Reaction temperature is too low	Ensure the reaction mixture is refluxed prior to addition of ethyl diazoacetate
	Product decomposed	Store in the refrigerator
Co‐elution during column chromatography	Starting with dichloromethane in the solvent system	Use neat hexane/heptane to elute excess COD before switching to 1:4 dichloromethane in heptane
	Column diameter is too small	Use a wider diameter column to minimize the height of the loading band

## Basic Protocol 2 SYNTHESIS OF (*endo*‐BICYCLO[6.1.0]NON‐4‐YN‐9‐YL)METHANOL 6a

This protocol (Fig. 2) describes the three‐step synthesis of (*endo*‐bicyclo‐[6.1.0]‐non‐4‐yn‐9)methanol **6a** from precursor **3a**. The first step involves reduction of the ester moiety to a primary alcohol, affording **4a** as an oil that can be used without further purification. Standard bromination of this material provides **5a** as high‐purity crystals after recrystallization in heptane. Elimination of the dibromide using potassium *tert*‐butoxide affords the stained alkyne **6a** as a white solid after column chromatography. The compounds were prepared with modifications to literature procedures (Dommerholt et al., 2010; Li, Liu, & Dong, 2017). Using the same procedures, beginning with *exo*‐**3b**, *exo*‐alcohol **6b** can be prepared in comparable yields.

### Materials

*endo*‐ethyl bicyclo[6.1.0]non‐4‐ene‐9‐carboxylate **3a** (from Basic Protocol 1)
Tetrahydrofuran (ACP Chemicals)
Lithium aluminum hydride (1 M in THF, Sigma Aldrich)
Anhydrous magnesium sulfate
Dichloromethane (ACP Chemicals)
Bromine (Sigma Aldrich)
Sodium thiosulfate (Sigma Aldrich)
Sodium chloride (Sigma Aldrich)
Potassium *tert*‐butoxide (20 wt.% solution in THF, ThermoFisher)
Ammonium chloride (Sigma Aldrich)
Heptane (ACP Chemicals)
Ethyl acetate (ACP Chemicals)

Round bottom flasks, 100 ml, 25 ml, and 250 ml
Ice bath
Rotary evaporator
NMR spectrometer
Separatory funnel
Chromatography column

### Reduction of ethyl endo‐bicyclo‐[6.1.0]‐non‐4‐ene‐9‐carboxylate 3a

1 Charge a 100‐ml round bottom flask with a magnetic stir bar, purified ester substrate **3a** (from Basic Protocol 1 above, 1.040 g, 5.35 mmol), and tetrahydrofuran (5 ml).
2 Cool the flask in an ice bath and bubble nitrogen through the solution for 10 min.
3 Add lithium aluminum hydride (1 M, 10.5 ml, 10.5 mmol) dropwise at 0°C with moderate stirring.
4 Allow the flask to slowly warm to room temperature and then stir for an additional hour.
5 Place the flask in an ice bath and quench the reaction by adding a few drops of cold water.
6 Add anhydrous magnesium sulfate to absorb the water.
7 Filter the suspension and concentrate the resulting solution using a rotary evaporator.
8 The crude product **4a**, a pale‐yellow oil, is used in the next step without further purification. This should be done as soon as possible (e.g., later the same day or the next day) to maximize the yield of **5a**.
  Crude yield 92%. ^1^H NMR (CDCl_3_, 400 MHz): δ 5.67‐5.59 (m, 2H), 3.72 (d, *J* = 7.6 Hz, 2H), 2.41‐2.32 (m, 2H), 2.14‐2.05 (m, 2H), 2.03‐1.94 (m, 2H), 1.62‐1.53 (m, 2H), 1.33 (s, 1H), 1.17‐1.09 (m, 1H), and 1.05‐0.96 (m, 2H) ppm.

### Bromination of (endo‐bicyclo[6.1.0]non‐4‐en‐9‐yl)methanol 4a

9 Charge a 25‐ml round bottom flask with a magnetic stir bar, alkene substrate **4a** (747.0 mg, 4.91 mmol), and dichloromethane (5 ml).
10 Cool the flask in an ice bath.
11 Add bromine (0.25 ml, 4.88 mmol) dropwise at 0°C with moderate stirring.
12 Allow the flask to slowly warm to room temperature and then stir for an additional hour in the dark.
13 Quench the reaction with 10 weight % sodium thiosulfate solution.
14 Transfer to a separatory funnel and extract the contents with dichloromethane (3 × 25 ml).
15 Wash the combined organic layers with saturated sodium chloride solution (25 ml).
16 Dry over anhydrous magnesium sulfate, filter, and concentrate on the rotary evaporator.
17 Recrystallize the off‐white solid in hot heptane to afford dibromide **5a** as white needles.
18 Yield 92%. ^1^H NMR (CDCl_3_, 400 MHz): δ 4.87‐4.79 (m, 2H), 3.77 (d, *J* = 7.3 Hz, 2H), 2.75‐2.62 (m, 2H), 2.32‐2.23 (m, 1H), 2.19‐2.13 (m, 1H), 1.98‐1.87 (m, 2H), 1.70‐1.59 (m, 2H), 1.58 (bs, 1H), and 1.25‐1.06 (m, 3H) ppm. ^13^C NMR (CDCl_3_, 100 MHz): δ 59.8, 56.3, 53.4, 35.1, 22.0, 20.2, 20.1, 19.1, and 17.3 ppm.
19 Dried and purified **5a** can be stored indefinitely if sealed under nitrogen or argon, stored cold (4°C), and protected from light.

### Elimination of (endo‐4,5‐dibromobicyclo[6.1.0]nonan‐9‐yl)methanol 5a

20 Charge a 250‐ml round bottom flask with a magnetic stir bar, dibromide substrate **5a** (1.950 g, 6.25 mmol), and tetrahydrofuran (60 ml).
21 Cool the flask in an ice bath and bubble nitrogen through the solution for 15 min.
22 Add potassium *tert*‐butoxide (20 wt.%, 11.5 ml, 19.1 mmol) at 0°C with moderate stirring.
23 Heat the reaction mixture to reflux and stir for 2 hr.
24 Quench any excess base with a saturated solution of ammonium chloride (50 ml).
25 Remove the tetrahydrofuran on the rotary evaporator.
26 Transfer the contents to a separatory funnel.
27 Add distilled water (50 ml) and extract the contents with dichloromethane (3 × 50 ml).
28 Wash the combined organic layers with saturated sodium chloride solution (50 ml).
29 Dry over anhydrous magnesium sulfate, filter, and concentrate on the rotary evaporator.
30 Purify the material by column chromatography, eluting with ethyl acetate in dichloromethane (2:98 → 5:95) to afford product **6a** as a white solid.
31 Yield 42%. ^1^H NMR (CDCl_3_, 400 MHz): δ 3.74 (d, *J* = 7.9 Hz, 2H), 2.34‐2.19 (m, 6H), 1.66‐1.56 (m, 2H), 1.45 (s, 1H), 1.39‐1.30 (m, 1H), and 0.99‐0.90 (m, 2H) ppm. ^13^C NMR (CDCl_3_, 100 MHz): δ 99.0, 59.9, 29.1, 21.6, 21.4, and 20.1 ppm (Table 2).

**Table 2:** Troubleshooting Guide for the Preparation of BCN

Problem	Possible cause	Solution
Gel forms during filtration	By‐products of the lithium aluminum hydride (LAH) quench	Use 1 M HCl solution (4‐5 equivalents relative to LAH) to quench, followed by standard aqueous workup
Product is obtained as a yellow‐brownish oil after bromination	Impurities carried over from previous reaction steps	Recrystallize the material in heptane (may require refrigeration to induce a solid)
	Insufficient bromine used and leftover starting material is remaining	Repeat the reaction ensuring enough bromine is used to convert the remaining starting material
Emulsion forms during the workup of BCN	Excess tetrahydrofuran	Remove the tetrahydrofuran on the rotary evaporator until only the ammonium chloride solution remains
Streaking during column chromatography	Poor solubility of the compound in ethyl acetate/heptane	Use solvent system consisting of ethyl acetate in dichloromethane to obtain the product in fewer fractions
Unable to see on TLC	UV‐inactive	Use potassium permanganate staining to visualize the compound on TLC
Poor BCN yield	Impure starting material	Ensure dibromide is used as white crystals
	Quality of the potassium *tert*‐butoxide	Use a fresh bottle of potassium *tert*‐butoxide or more equivalents
	Product decomposed	Store under N_2_ in the freezer
Messy NMR spectrum	Minor impurities	Recrystallize in heptane

## Basic Protocol 3 SYNTHESIS OF *endo*‐BCN‐E‐BCN 1

This protocol (Fig. 2) describes the two‐step synthesis of BCN‐E‐BCN **1** (Fig. 1C) from precursor **6a**. The first step involves converting the alcohol to a carbonate functionality with a labile *p*‐nitrophenoxy leaving group to obtain compound **7a**. One equivalent of ethylenediamine is then reacted with two equivalents of BCN electrophile **7a** to generate the bis‐BCN molecular probe **1** as a white solid. The compounds were prepared with modifications to previously published procedures (McGarry et al., 2016).

### Materials

*endo*‐bicyclo[6.1.0]non‐4‐yn‐9‐yl)methanol **6a** (from Basic Protocol 2)
Dichloromethane (ACP Chemicals)
Anhydrous pyridine (Sigma Aldrich)
*p*‐Nitrophenyl chloroformate (Sigma Aldrich)
Ammonium chloride (Sigma Aldrich)
Sodium chloride (Sigma Aldrich)
Magnesium sulfate
Ethyl acetate (ACP Chemicals)
Heptane (ACP Chemicals)
Acetonitrile
*N,N*‐dimethylformamide
Triethylamine (Sigma Aldrich)
Ethylenediamine

Round bottom flasks, 50 ml, 25 ml
Magnetic stir bar
Separatory funnel
Rotary evaporator
Chromatography column
NMR spectrometer
High‐resolution electrospray mass spectrometer

### Coupling of 6a to p‐nitrophenyl chloroformate

1 Charge a 50‐ml round bottom flask with a magnetic stir bar, **6a** (200.0 mg, 1.33 mmol), and dichloromethane (25 ml).
2 Bubble nitrogen through the solution for 15 min.
3 Add anhydrous pyridine (0.27 ml, 3.35 mmol) followed by *p*‐nitrophenyl chloroformate (335.0 mg, 1.66 mmol).
4 Stir at room temperature for 1 hr.
5 Quench the reaction with a saturated solution of ammonium chloride (25 ml).
6 Transfer to a separatory funnel and extract the contents with dichloromethane (3 × 25 ml).
7 Wash the combined organic layers with saturated sodium chloride solution (50 ml).
8 Dry over magnesium sulfate, filter, and concentrate on the rotary evaporator.
9 Purify the material by column chromatography, eluting with 5:95 ethyl acetate in heptane to afford product **7a** as a white solid.
10 Yield 78%. ^1^H NMR (CDCl_3_, 400 MHz): δ 8.30‐8.26 (m, 2H), 7.41‐7.37 (m, 2H), 4.42 (d, *J* = 8.2 Hz, 2H), 2.37‐2.20 (m, 6H), 1.66‐1.56 (m, 2H), 1.54‐1.49 (m, 1H), and 1.11‐1.02 (m, 2H) ppm.

### Preparation of molecular probe 1

11 Charge a 25‐ml round bottom flask with a magnetic stir bar, BCN electrophile **7a** (315.0 mg, 1.00 mmol), and 1:1 acetonitrile/*N,N*‐dimethylformamide (12 ml).
12 Add triethylamine (0.28 ml, 2.00 mmol) and then a solution of ethylenediamine in *N,N*‐dimethylformamide (1 M, 0.50 ml, 0.50 mmol).
13 Stir at room temperature for 1 hr.
14 Concentrate on the rotary evaporator.
15 Purify the material by column chromatography, eluting with 2:3 ethyl acetate in heptane to afford **1** as a white solid.
16 Yield 58%. ^1^H NMR (CDCl_3_, 400 MHz): δ 5.01 (bs, 2H), 4.15 (d, *J* = 8.0 Hz, 4H), 3.32 (s, 4H), 2.34‐2.17 (m, 12H), 1.63‐1.52 (m, 6H), and 0.97‐0.92 (m, 4H) ppm. [HRMS‐ESI] *m/z*: [M+H]^+^ calculated for C_24_H_32_N_2_O_4_: 413.2440, found: 413.2435 (Table 3).

**Table 3:** Troubleshooting Guide for the Preparation of BCN‐E‐BCN

Problem	Possible cause	Solution
Poor BCN electrophile yield	*p*‐nitrophenyl chloroformate added too fast at room temperature	Add slowly or on ice
	Material decomposed on silica gel	Wet‐load the material using a minimal amount of dichloromethane (diluting with heptane if necessary)
	Product decomposed	Store under N_2_ in the refrigerator
Poor BCN‐E‐BCN yield	Excess ethylenediamine used	Ensure only 1 equivalent of ethylenediamine is used relative to the BCN electrophile
	Order of addition	Add 1 M solution of ethylenediamine to the substrate and not the substrate to the ethylenediamine solution
	Product decomposed	Store under N_2_ in the freezer
Unable to see on TLC	UV‐inactive	Use potassium permanganate staining to visualize the compound on TLC

## Basic Protocol 4 BCN‐E‐BCN TREATMENT OF RECOMBINANT WILD‐TYPE AND CYSTEINE‐DEFICIENT MUTANT COFILIN PROTEIN

The following protocol describes steps to test the utility of synthesized BCN‐E‐BCN as an oxidation probe and its ability to detect oxidation of cysteine residues in a rapid and highly sensitive manner when compared to dimedone. Purified recombinant wild‐type cofilin and a cysteine‐deficient form of cofilin, in which two redox‐sensitive cysteines were mutated to alanine residues (C139A/C147A) to render it resistant to hydrogen peroxide–mediated oxidation, were exposed to differing amounts of hydrogen peroxide *in vitro*, and then labeled with BCN‐E‐BCN. A copper‐free click reaction with azide‐PEG3‐biotin was used to label the free end of the BCN‐E‐BCN probe. Ice‐cold acetone quenched the excess unreacted azide‐PEG3‐biotin and precipitated the protein. The resulting protein pellet was resuspended in the polyacrylamide gel electrophoresis (PAGE) loading buffer to enable analysis through western blotting, as described in Basic Protocol 6.

### Materials

2 mg/ml recombinant wild‐type (WT) cofilin in TBS (see Support Protocol 1)
2 mg/ml recombinant C139A/C147A cofilin in TBS (see Support Protocol 1)
Hydrogen peroxide, 30% (Fisher Chemical H325500)
DI water
100 µM BCN‐E‐BCN suspended in 100% DMSO (see Basic Protocol 3)
4 mM dimedone suspended in 100% DMSO (Sigma‐Aldrich D153303)
4× LDS (lithium dodecyl sulfate) sample buffer (Invitrogen B0007)
2‐Mercaptoethanol (Sigma‐Aldrich 444203‐250ML)
50 mM stock of azide‐PEG3‐biotin resuspended in 100% DMSO (Sigma‐Aldrich 762024)
Acetone ACS grade (Caledon 1200‐1‐40)

Microcentrifuge tubes
Micropipettes
Tube rotator
Temperature‐controlled microcentrifuge

### Preparation of protein samples and addition of BCN‐E‐BCN

1 Prepare 2 sets of the protein sample, with each set having 3 microcentrifuge tubes containing 10 µg of cofilin WT and 3 microcentrifuge tubes containing 10 µg of cofilin C139A/C147A in duplicate; the total number of tubes would be 12 (6 tubes for each set).
  5 µl of protein is needed per sample if using a protein stock concentration of 2 mg/ml.
2 Set aside the 3 tubes containing cofilin WT and add 10 µl of DI water to 1 tube, 10 µl of a 0.2 mM stock of H_2_O_2_ made in DI water to the next tube (final concentration of 0.1 mM H_2_O_2_), and 10 µl of a 2 mM stock of H_2_O_2_ made in DI water to the third tube (final concentration of 1 mM H_2_O_2_).
3 Repeat the previous step for the tubes containing cofilin C139A/C147A, ensuring that each set has 3 tubes of cofilin WT that have been treated with either DI water or H_2_O_2_ and 3 tubes of cofilin C139A/C147A that have been treated with either DI water or H_2_O_2_.
4 Add 5 µl of BCN‐E‐BCN (final concentration of 25 µM) to one set of samples and 5 µl of dimedone (final concentration of 1 mM) to the other set and incubate at room temperature for 20 min.
  The final sample volume should be 20 µl.
5 After the incubation, add boiling buffer (1× LDS with 10% 2‐mercaptoethanol) to the dimedone‐labeled samples.
6 Heat the dimedone‐labeled samples at 95°C for 5 min and proceed with western blotting, as described in Basic Protocol 6.
7 Continue to step 8 with the BCN‐E‐BCN samples.

### Copper‐free click reaction of BCN‐E‐BCN‐labeled residues and azide‐PEG3‐biotin

8 Add 1 mM azide‐PEG3‐biotin to each sample.
9 Incubate the samples on a tube rotator at room temperature for 1 hr.
10 Add three times the sample volume (60 μl) of chilled acetone to each sample.
11 Vortex each sample for a few seconds and cool to −80°C for 1 hr.
12 Spin the samples in a 4°C microcentrifuge at 16,000 RCF for 10 min.
13 Remove the supernatant, taking care to not disturb the pellet.
14 Leave the sample tubes uncapped for 5 to 10 min to allow excess acetone to evaporate.
  Do not let the sample dry out for too long; otherwise, the pellet will become difficult to resuspend.
15 Resuspend each pellet in 50 µl of boiling buffer (1× LDS with 10% 2‐mercaptoethanol).
16 Heat each sample at 95°C for 5 min.
17 Proceed with western blotting, as described in Basic Protocol 6 (Table 4).

**Table 4:** Troubleshooting Guide for BCN‐E‐BCN Treatment of Purified Recombinant Cofilin and Mutant Cofilin Protein

Problem	Possible cause	Solution
Difficulty resuspending protein pellet in boiling buffer	Pellet has been overdried after acetone removal	Carefully vortex sample to aid in resuspension, taking care not to overdry remaining samples

## Support Protocol 1 PURIFICATION OF RECOMBINANT WILD‐TYPE COFILIN AND C139A/C147A COFILIN

This protocol is based on the work by Cameron et al. (2015) that describes the purification of recombinant cofilin in both its wild‐type and C139A/C147A mutant forms for use in *in vitro* experiments, as described in Basic Protocol 4. Cameron et al. found that cofilin is oxidized at its C139 and C147 sites, rendering it inactive; therefore, the C139A/C147A mutant serves as a negative control in the oxidation assay described in Basic Protocol 4.

### Materials

*E. coli* BL21 (DE3) pLys cells (Agilent Technologies 200132)
Primers
  C139A: (5′‐GAA TTG CAA GCA AAC GCG TAC GAG GTC AAG GAC CGC‐3′)
  C147A: (5′‐GAG GTC AAG GAC CGC GCG ACC CTG GCA GAG AAG ‐3′)
pGEX‐KG‐hCofilin WT
QuikChange II XL Site‐Directed Mutagenesis Kit (Agilent Technologies cat no. 200521)
  PfuUltra High Fidelity DNA polymerase (2.5 U/µl)
  10× reaction buffer
  Dpn I restriction enzyme (10 U/µl)
  Oligonucleotide control primer #1 [34‐mer (100 ng/µl)] 5´ CCA TGA TTA CGC CAA GCG CGC AAT TAA CCC TCA C 3´
  Oligonucleotide control primer #2 [34‐mer (100 ng/µl)] 5´ GTG AGG GTT AAT TGC GCG CTT GGC GTA ATC ATG G 3´
  pWhitescript 4.5‐kb control plasmid (5 ng/µl)
  dNTP mix
  XL10‐Gold ultracompetent cells (yellow tubes)
  XL10‐Gold β‐mercaptoethanol mix (β‐ME)
  pUC18 control plasmid (0.1 ng/µl in TE buffer)
Luria Broth (LB)‐ampicillin agar plates (see recipe)
LB broth (Miller) (see recipe)
Ampicillin (Bioshop AMP201.5)
Isopropyl β‐d‐1‐thiogalactopyranoside (IPTG, Bioshop IPT001.5)
Bacterial lysis buffer (see recipe)
Glutathione sepharose beads (Sigma‐Aldrich GE17‐0756‐01)
Wash buffer 1 (see recipe)
Wash buffer 2 (see recipe)
1000 units thrombin suspended in 1 ml of DI water (Sigma‐Aldrich T4648‐1KU)
ρ‐aminobenzamidine beads (Sigma‐Aldrich A7155‐10ML)
Tris‐buffered saline (TBS): 50 mM Tris·HCl (pH 7.4) and 150 mM NaCl

Hot water bath
Cell spreaders
Shaking incubator
Spectrophotometer
High‐speed centrifuge
Centrifuge bottles, 1 L
Falcon tube, 50 ml
Separation column
Sonicator
Tube rotator

### Constructing the mutant C139A/C147A cofilin plasmid

1 The following forward and corresponding reverse primers were respectively used to create the C139A and C147A mutations in the pGEX‐KG‐hCofilin WT plasmid:
  C139A: (5′‐GAA TTG CAA GCA AAC GCG TAC GAG GTC AAG GAC CGC ‐3′)
  C147A: (5′‐GAG GTC AAG GAC CGC GCG ACC CTG GCA GAG AAG‐3′)
    Primers may differ depending on the mutagenesis kit used.
2 Both primers need to be used at the same time to create the C139A/C147A cofilin mutant.
3 Follow the manufacturer's instructions for the specific mutagenesis kit being used to produce full‐length plasmids.
4 Verify the desired mutations by sequencing the resulting pGEX‐KG‐CFL C139A/C147A plasmid.

### Transforming E. coli with pGEX‐KG‐CFL and pGEX‐KG‐CFL C139A/C147A

5 Thaw one 50‐µl aliquot of BL21 (DE3) *E. coli* for each plasmid on ice for 10 min.
6 Add 1 to 20 ng of pGEX‐KG‐CFL to one tube of *E. coli* and 1 to 20 ng of pGEX‐KG‐CFL C139A/C147A to the other tube of *E. coli*.
  Caution: Do not mix the tube contents.
7 Incubate the tubes on ice for 30 min.
8 Warm two LB‐ampicillin agar plates and a 1‐ml aliquot of LB broth (Miller) to 37°C during the waiting period, and set a hot water bath to warm the tubes to 42°C.
9 Heat shock the bacteria by placing tubes in the water bath at 42°C for 30 s once the initial incubation is over.
10 Immediately place the heat‐shocked tubes on ice for 2 min.
11 Add 450 µl of LB broth (Miller) from the previously warmed aliquot to each of the tubes.
12 Drop ∼200 µl of the diluted bacteria mixture on one LB‐amp agar plate each and gently spread using a cell spreader.
  Spread until the liquid mixture is evenly dispersed and the agar no longer feels wet and slick.
13 Place the plates in an incubator at 37°C to grow overnight.

### Inducing protein expression in E. coli cultures

14 Pick a colony of the transformed cells expressing either pGEX‐KG‐CFL WT or pGEX‐KG‐CFL C139A/C147A to create liquid starter cultures that are then incubated overnight at 37°C while shaking.
15 Dilute the overnight cultures the following morning at 1:10 in LB (Miller) containing 100 µg/ml ampicillin.
16 Grow the cultures in a shaking incubator set to 37°C until the OD_600_ reaches 0.6 to1.0, measured using the spectrophotometer.
17 Induce protein expression by adding 100 µM IPTG and continuing to incubate the cultures for 3 hr.
18 Pellet cells by spinning the culture down at 4000 RCF for 20 min at 4°C in 1‐L centrifuge bottles.

### Bacterial lysis

19 Discard the supernatant and resuspend the resulting pellet in 30 ml of bacterial lysis buffer.
20 Incubate the lysate in a 50‐ml Falcon tube on ice for 30 min.
21 Sonicate the lysate using the following settings:
*Amplitude: 25%*
*Time: 3 min*
*Pulse on: 15 s*
*Pulse off: 60 s*
22 Spin down the lysate at 20,000 RCF at 4°C for 30 min and set aside on ice.

### Preparation of glutathione sepharose beads

23 Use ∼300 µl of glutathione sepharose beads per 30 ml of lysate.
24 Resuspend beads with 1 ml of wash buffer 1; then spin down at 500 RCF in a 4°C chilled centrifuge and remove the supernatant to wash away residual storage ethanol.
25 Repeat wash step described above using wash buffer 2.
26 Combine the lysate and the washed beads and incubate the mixture overnight at 4°C on a tube rotator.

### Protein purification

*Perform each step of the purification process at 4°C unless otherwise specified*.

27 Pour the bead and lysate mixture into an uncapped separation column, allowing the liquid to flow out.
28 Wash the column with 10 bed volumes of chilled wash buffer 1 and then 5 bed volumes of chilled wash buffer 2. Allow the liquid to reach the top of the column bed and then cap the outflow.
29 Add 10 U (10 µl) of thrombin per mg of recombinant protein.
30 Incubate the mixture at 4°C overnight.
31 Remove the cap and collect the flow through. Wash the column with 1 bed volume of chilled wash buffer 1.
32 Resuspend 100 µl of ρ‐aminobenzamidine beads in 500 µl TBS; then spin down at 500 RCF in a 4°C centrifuge and remove the supernatant to wash away residual storage ethanol. Repeat the wash twice more.
  ρ‐aminobenzamidine binds and inhibits proteases such as thrombin. When coupled to beads, it is able to bind thrombin once it has cleaved the GST tag from the protein. Afterward, the protein is free to flow out, whereas thrombin remains bound to the beads.
33 Add the newly washed ρ‐aminobenzamidine beads to the thrombin and protein mixture and incubate on a tube rotator at 4°C for 30 min.
34 Spin down the mixture for 2 min in a 4°C centrifuge and collect the supernatant containing cleaved protein cofilin that has been separated from thrombin.
35 Determine protein yield by carrying out a Bradford assay.
  The typical yield from a 2‐L culture is ∼6 mg of protein for both WT and mutant cofilin. The protein is then aliquoted and stored at 2 mg/ml in TBS (Table 5).

**Table 5:** Troubleshooting Guide for Purification of Recombinant Wild‐Type and C139A/C147A Cofilin

Problem	Possible cause	Solution
No colony growth after bacteria transformation	Incorrect antibiotic plate used	Verify the plasmid resistance gene and plates
	Inefficient transformation	Re‐optimize PCR conditions and parameters
No overnight culture growth	Incorrect antibiotic present	Verify the plasmid resistance gene and use the correct antibiotic
	Using a colony from an expired plate	Re‐transform *E. coli* and use a fresh colony
No protein present in western blots	Protein was not cleaved by thrombin and is still bound to glutathione sepharose beads	Optimize and adjust wash buffer composition and pH. Use freshly prepared thrombin. Re‐cleave protein from beads
	Protein was lost in washing stage of purification	Start over, keeping samples at each stage of the procedure for monitoring

## Basic Protocol 5 BCN‐E‐BCN LABELING IN LIVE CELLS

This protocol describes the optimization process of the BCN‐E‐BCN dose and treatment time to label oxidized cysteines in live cells, specifically HEK 293 human embryonic kidney cells. BCN‐E‐BCN is cell permeable and is able to rapidly react with cysteine sulfenic acids. After lysis and sample normalization, the protocol converges with Basic Protocol 4, where azide‐PEG3‐biotin is added to the labeled cell lysates to react with the free end of the BCN‐E‐BCN probe bound to the cysteine sulfenic acid of the oxidized protein in a copper‐free click reaction. The excess azide‐PEG3‐biotin is quenched, and the proteins are precipitated using ice‐cold acetone. The resulting protein pellet is resuspended in loading buffer. The samples are analyzed by western blot analysis for visualizing the BCN‐E‐BCN‐labeled residues.

### Materials

HEK 293 human embryonic kidney cells
DMEM high glucose with L‐glutamine and sodium bicarsbonate, supplemented with 10% fetal bovine serum (FBS) and 1% penicillin‐streptomycin (P/S) (Sigma‐Aldrich)
DMSO (Fisherbrand BP231100)
10 mM BCN‐E‐BCN resuspended in 100% DMSO (see Basic Protocol 3)
Phosphate‐buffered saline (PBS, pH 7.4)
Lysis buffer (see recipe)
Pierce BCA Protein Assay Kit (Thermo Scientific cat no. 23227)
  BCA Reagent A, 500 ml
  BCA Reagent B, 25 ml
  Albumin Standard Ampules, 2 mg/ml, 10 × 1 ml
50 mM stock of azide‐PEG3‐biotin resuspended in 100% DMSO (Sigma‐Aldrich 762024)
Acetone ACS grade (Caledon 1200‐1‐40)

Micropipettes
Humidified cell incubator at 37°C and 5% CO_2_ concentration
Microcentrifuge tubes
Cell scrapers
Temperature‐controlled microcentrifuge

### Optimizing BCN‐E‐BCN treatment in HEK 293 cells

1 Seed 3 million HEK 293 cells in DMEM in 7 10‐cm tissue culture dishes (4 dishes for dose optimization and 3 dishes for treatment time optimization).
  Dose optimization samples will be treated with 0, 25, 50, and 100 µM BCN‐E‐BCN.
  Treatment time optimization samples will be subjected to BCN‐E‐BCN incubation for 0, 5, and 15 min.
2 Treat the dosage optimization dishes with a DMSO vehicle control and BCN‐E‐BCN with final concentrations of 25, 50, and 100 µM the next day (when cells have reached ∼70% confluency).
  Gently rock each plate after adding BCN‐E‐BCN.
3 Incubate the dosage optimization dishes for 15 min in a humidified incubator at 37°C and 5% CO_2_ concentration.
4 Add BCN‐E‐BCN with a final concentration of 100 µM to the treatment time optimization dishes when cells have reached 70% confluency.
5 Incubate 1 treatment time optimization dish for 5 min and another dish for 15 min in the humidified incubator at 37°C and 5% CO_2_ concentration. The third dish (0 min incubation) is immediately lysed (proceed to step 7) after BCN‐E‐BCN treatment.
6 Remove the cells from the incubator after their respective incubation times and proceed with the protocol described in steps 7 to 19.

### HEK 293 cell lysis and sample normalization

7 Place plates on ice.
8 Aspirate medium.
9 Gently wash each plate with 5 ml of ice‐cold PBS (pH 7.4) twice.
  Ensure that PBS is dispensed against the wall of the plate to minimize cell detachment.
10 Remove remaining PBS with a micropipette.
11 Dispense 1 ml of ice‐cold lysis buffer on each plate and rock.
12 Scrape the plate thoroughly with a cell scraper.
13 Tilt the plate and transfer the lysis buffer containing the scraped cells into a chilled and labeled microcentrifuge tube using a micropipette.
14 Leave the samples to rotate on a tube rotator at 4°C for 20 min.
15 Spin down samples in a 4°C microcentrifuge at 16,000 RCF for 10 min.
16 Remove the samples and transfer the lysate into new chilled microcentrifuge tubes, taking care to not to disturb the pellet.
17 Perform a BCA assay on the obtained lysates and normalize the protein amounts in the samples to 1 mg/ml. Keep samples on ice.
  The typical protein yield is ∼2 mg/ml, which would require dilution to the target yield of 1 mg/ml.
18 Add 1 mM of azide‐PEG3 to each sample (usually 10 to 50 µg of protein lysate).
19 Proceed with steps 5 to 13, as described in Basic Protocol 4 (copper‐free click reaction of BCN‐E‐BCN‐labeled residues and azide‐PEG3‐biotin) (Table 6).

**Table 6:** Troubleshooting Guide for BCN‐E‐BCN Labeling in Live Cells

Problem	Possible cause	Solution
Low protein lysate yields	High cell death/loss of viable cells	Optimize BCN‐E‐BCN treatment time and dose for different cell types
Difficulty resuspending protein pellet in boiling buffer	Pellet has been overdried after acetone removal	Carefully vortex sample to aid in resuspension, taking care not to overdry remaining samples

## Basic Protocol 6 WESTERN BLOTTING AND VISUALIZATION OF BCN‐E‐BCN‐LABELED SAMPLES

Once the oxidized protein samples have been labeled with BCN‐E‐BCN and reacted with azide‐PEG3‐biotin, they can be visualized by western blotting. This protocol describes the process of running an SDS‐PAGE gel, transfer of the protein samples to nitrocellulose membranes, western blotting, and subsequent imaging using fluorescently tagged streptavidin.

### Materials

BCN‐E‐BCN‐labeled samples obtained from Basic Protocols 4 and 5
Bolt^™^ 4 to 12%, Bis‐Tris, 1.0 mm 12‐well Mini Protein Gel (Invitrogen NW04122BOX)
20× MES running buffer (Invitrogen B000202)
Transfer buffer (see recipe)
TBS‐Tween (TBST): TBS with 0.1% Tween 20 detergent
Bovine serum albumin (BSA, Bioshop ALB005.250)
Anti‐cysteine sulfenic acid rabbit polyclonal antibody (MilliporeSigma 07‐2139‐I‐25UL)
Anti‐GAPDH mouse monoclonal antibody (Sigma‐Aldrich G8795‐100UL)
IRDye^®^ 680RD Streptavidin (LI‐COR 925‐68079)
IRDye^®^ 680RD Goat anti‐Rabbit IgG Secondary Antibody (LI‐COR 926‐68071)
IRDye^®^ 800CW Goat anti‐Mouse IgG Secondary Antibody (LI‐COR 926‐32210)
Precision Plus Protein Dual Color Standard molecular weight ladder (Bio‐Rad 1610374)

SDS‐PAGE tank
Container
Transfer tank with cassettes and sponges
Filter paper
Nitrocellulose membranes
Roller
Ice pack
Rocker
Sealable pouch
LI‐COR Odyssey CLx imaging system (LI‐COR)

### Running SDS‐PAGE

1 Load 10 µg of each sample (obtained from Basic Protocols 4 and 5) and 5 µl of protein molecular weight ladder in a 4 to 12% gradient SDS PAGE in a tank containing 1× MES running buffer.
2 Set the SDS‐PAGE to run at 80 V for 15 min, and then increase the voltage to 120 V.
3 Let the SDS‐PAGE run for approximately 1 hr, stopping when the dye front reaches the bottom of the gel.

### Transfer of proteins to nitrocellulose membrane

4 Set up a wide container containing chilled transfer buffer.
5 Submerge four pieces of filter paper, one nitrocellulose membrane, the transfer cassette, and sponges in transfer buffer.
6 Remove the SDS‐PAGE from the tank and rinse it well in DI water; then crack open the cassette and trim the wells and bottom strip of the gel.
7 Create the transfer sandwich using the following order: black side of the cassette, sponge, two pieces of filter paper, SDS‐PAGE, nitrocellulose membrane, two pieces of filter paper, sponge, and top of the cassette.
  Using a small roller, make sure to gently, but firmly, roll out any air bubbles after placing filter papers on top of the nitrocellulose membrane.
8 Place the transfer cassette into the tank and carefully pour in the cold transfer buffer, making sure to avoid pouring directly on top of the cassette.
9 Add an ice pack and top up with transfer buffer until it reaches the fill line in the tank.
10 Run the transfer at 100 V for 75 min.
11 Remove the membrane and wash it for 5 min in TBST at room temperature on a rocker.
12 Block the membrane in 3% BSA made in TBST for 1 hr at room temperature or overnight at 4°C on a rocker.

### Imaging of oxidized proteins using fluorescently labeled streptavidin

13 Make a solution using 3% BSA in TBST containing primary antibodies of interest if applicable.
  Membranes containing samples from Basic Protocol 4 could be blotted using a 1:1000 dilution of anti‐cysteine sulfenic acid rabbit polyclonal antibody. Membranes containing samples from Basic Protocol 5 could be blotted using a 1:1000 dilution of anti‐GAPDH mouse monoclonal antibody to ensure equal loading.
14 Incubate the membrane in the antibody solution in a sealed pouch for 1 hr at room temperature or overnight at 4°C on a rocker.
15 Remove the primary antibody solution and wash the membrane in a container three times for 5 min per wash using TBST.
  Make a mixture of 3% BSA in TBST containing a 1:20,000 dilution of IRDye 680RD streptavidin; add it to the membrane and incubate at room temperature for 1 hr on a rocker. Ensure the membrane is protected from light exposure during this period.
  If working with a membrane from Basic Protocol 4, also add IRDye^®^ 680RD Goat anti‐Rabbit IgG Secondary Antibody to visualize the dimedone signal. If working with a membrane using samples from Basic Protocol 5, also add IRDye^®^ 800CW Goat anti‐Mouse IgG Secondary Antibody diluted to 1:20,000 to the mixture.
16 Remove the streptavidin solution and wash the membrane four times for 7 min per wash.
  The membrane should still be protected from light during the washing period.
17 Image the membrane on a LI‐COR Odyssey CL‐x (Table 7).

**Table 7:** Troubleshooting Guide for Western Blotting and Visualization of BCN‐E‐BCN Labeled Samples

Problem	Possible cause	Solution
Smeared/spotty image	Residual TBST on the membrane	Increase washing time
Bubbles on image	Improper transfer process	Carefully and thoroughly roll out bubbles for each layer of the transfer sandwich
Faint/no bands appearing	Too little azide‐PEG3‐biotin added	Optimize azide‐PEG3‐biotin treatment dose and incubation time for each experiment type
	Too little streptavidin added	Decrease streptavidin dilution
Blown out signal for streptavidin blot	Too much azide‐PEG3‐biotin added	Optimize azide‐PEG3‐biotin treatment dose and incubation time for each experiment type
	Too much streptavidin added	Increase streptavidin dilution or shorten incubation time

## REAGENTS AND SOLUTIONS

### Bacterial lysis buffer (50 ml)

- 1× TBS
- 2× complete protease inhibitor
- 3 mM DTT
- 1 mg/ml lysozyme
- Discard solution after use

### HEK 293 lysis buffer (10 ml)

- 7.4 ml H_2_O
- 1 ml 10× TBS, pH 7.6
- 1 ml 10% triton X‐100 solution
- 20 µl 0.5 M EDTA
- 400 µl 0.5 M sodium fluoride
- 200 µl 1 M β‐glycerophosphate
- 1× complete protease inhibitor tablet

### Hydrogen peroxide (H_2_O_2_), 0.2 mM stock (100 µl)

- 2 µl hydrogen peroxide, 30% (Fisher Chemical H325500)
- 80 µl DI water

### Hydrogen peroxide (H_2_O_2_), 2 mM stock (100 µl)

- 2 µl hydrogen peroxide, 30% (Fisher Chemical H325500)
- 80 µl DI water

### Luria broth (LB) Miller (1 L)

- 10 g tryptone
- 5 g yeast extract
- 10 g NaCl
- 500 ml H_2_O to initially dissolve everything
- Top up to 1 L after all components have been dissolved
- Autoclave and store at room temperature for up to 3 months

### LB‐ampicillin agar plates (1 L)

- 10 g tryptone
- 5 g yeast extract
- 10 g NaCl
- 15 g agar
- Ampicillin (to 100 µg/ml concentration)
- 500 ml H_2_O to initially dissolve everything
- Top up to 1 L after all components have been dissolved
- Autoclave and store at room temperature for up to three months until ready to pour plates

### 1× MES running buffer (1 L)

- 50 ml 20× MES running buffer (Invitrogen B000202)
- 950 ml DI water

### 10× TBS, pH 7.6 (1 L)

- 24 g tris base
- 88 g NaCl
- 800 ml H_2_O to initially dissolve tris base and NaCl
- Adjust to pH 7.6 using concentrated HCl and then top up to 1 L with H_2_O
- Store at room temperature for up to 1 year

### 1× TBST (1 L)

- 1 L 1× TBS
- 1 ml Tween 20
- Store at room temperature for up to 1 year

### Transfer buffer (1 L)

- 3 g tris base
- 14.3 g glycine
- 800 ml H_2_O
- 200 ml methanol
- Store at 4°C for up to 3 months

### Tris buffer solution, 50, mM, pH 8.0 (50 ml)

- 0.303 g tris base
- 40 ml H_2_O to initially dissolve tris base
- Adjust to pH 8.0 using concentrated HCl and then top up to 50 ml with H_2_O
- Store up to 1 year at room temperature

### Wash buffer 1 (1 L)

- 1× TBS
- 3 mM DTT
- 1 mM EDTA

### Wash buffer 2 (1 L)

- 1× TBS
- 1 mM MgCl_2_
- 1 mM CaCl_2_
- 3 mM DTT

## COMMENTARY

### Background Information

As the collective view of oxidation within cells has evolved from it solely being a source of cellular damage to also being an important regulatory mechanism, the need for molecules to identify oxidized proteins, particularly cysteine sulfenic acids, has increased. Dimedone‐based probes have a history of use (Forman et al., 2017; Nelson et al., 2010; Paulsen & Carroll, 2009; Qian et al., 2012), but dimedone requires relatively long treatment times due to its slow reaction kinetics, which can lead to cysteine over‐oxidation from the sulfenic acid state to sulfinic and sulfonic acids when ROS levels are high (Heppner et al., 2018). There have been efforts to improve the utility of dimedone, including the addition of biotin to increase its functionality (Charles et al., 2007).

Although these types of modifications were improvements, we developed BCN‐E‐BCN as a simpler solution for the labeling of cysteine sulfenic acids, and this also has a number of useful advantages. As a bifunctional probe, BCN‐E‐BCN contains two highly reactive strained cyclooctynes that will rapidly react with cysteine sulfenic acids (McGarry et al., 2016). In addition to the greater reactivity, BCN‐E‐BCN is cell permeable, adding to its utility (McGarry et al., 2016). Once one BCN has reacted with the cysteine sulfenic acid, the other BCN remains available for labeling. For example, azide‐PEG3‐biotin can react with the free BCN in a copper‐free click reaction for enrichment and detection. The azide group reacts rapidly with the cyclooctyne group, while the biotin enables easy detection of oxidized proteins through western blotting by taking advantage of the high‐affinity binding of streptavidin to biotin.

The ability of BCN‐E‐BCN to be used for labeling live cells and purified protein samples demonstrates its versatility as a probe. Although it is still a relatively new probe that has not yet been widely utilized, there is great potential for the mainstream use of BCN‐E‐BCN for investigating oxidized cysteines. ROS have been shown to be important second messengers in cells, having a strong influence on signaling pathways, for example *via* the regulation of protein tyrosine phosphatases (Denu & Tanner, 1998) and receptor tyrosine kinases (Sag et al., 2013). Cancer cells may take advantage of these signaling pathways to increase their proliferation, migration, and survival (de Sá Junior et al., 2017; Ishikawa et al., 2008; Kim, Kim, Koh, Ho, & Lee, 2006), making the understanding of these and related pathways through the development of oxidation probes like BCN‐E‐BCN all the more important.

### Critical Parameters

**Basic Protocol** **1** **—**The copper catalyst should be dried beforehand by placing the solid in an open 20‐ml scintillation vial and then heating over a Bunsen burner until the color transitions to white. The rate of ethyl diazoacetate addition should be controlled to prevent the reaction mixture from boiling over into the condenser. Following rotary evaporation of the solution after hot filtration, hexane can be used to assist the transfer of the material to the silica column. Heptane can be used in place of hexane for material transfers and during column chromatography. Excess **2** (visualized by potassium permanganate staining) should be eluted using neat hexane, followed by transition to 1:4 dichloromethane in hexane to elute the diastereomers *endo*‐**3a** and *exo‐* **3b** (Fig. 2).

**Basic Protocol** **2** **—**In instances where a gel forms during the filtration, 1 M HCl should be used in place of distilled water followed by standard aqueous workup. This involves extraction with an appropriate organic solvent (diethyl ether or ethyl acetate). The combined organic layers are then washed with saturated sodium bicarbonate and sodium chloride solutions, dried over magnesium sulfate, filtered, and concentrated *in vacuo*.

For the subsequent elimination step, use of **5a** as an off‐white or a white solid is highly recommended. When carrying over a yellow‐brownish oil, the reaction yields have been consistently poor.

The tetrahydrofuran should be removed prior to workup of crude **6a** to avoid the formation of an emulsion. Potassium *tert*‐butoxide from Sigma Aldrich (1 M in tetrahydrofuran) has also been used successfully if satisfying the requirement of three equivalents (relative to the substrate). Alternatively, the material can be purified using 1:5 ethyl acetate in heptane. For the *exo*‐isomer **6b**, 2:3 ethyl acetate in heptane or 5:95 ethyl acetate in dichloromethane can be used. In the event of an oil, the material can be recrystallized in heptane. Refrigeration is sometimes required to induce formation of a white solid. The product is susceptible to decomposition and should be stored under nitrogen in the freezer.

**Basic Protocol** **3** **—**The *p*‐nitrophenyl chloroformate should be added slowly to avoid generation of large amounts of heat. Alternatively, it can be added on ice and then stirred at room temperature. The material should not be dried on silica gel prior to column chromatography due to the lability of the *p*‐nitrophenyl group. For the *exo*‐isomer **7b**, ethyl acetate in heptane (5:95 → 10:90) can be used for purification.

A solution of the nucleophile can be prepared by diluting ethylenediamine (0.67 ml, 10 mmol) in a 10‐ml volumetric flask with *N,N*‐dimethylformamide, followed by storing over 4Å molecular sieves. In the workup, heptane can be used to remove the *N,N*‐dimethylformamide azeotropically, if necessary. The material can be visualized on a TLC plate with potassium permanganate staining.

**Basic Protocol** **4** **—**The protocol parameters for biotinylation of BCN‐E‐BCN bound to cysteine sulfenic acids have been optimized for the use of azide‐PEG3‐biotin. It is possible to use other biotin‐based molecules; however, concentrations and incubation times would need to be determined empirically. An hour of incubation at −80°C following the addition of ice‐cold acetone is effective; however, if there are time constraints, then the incubation can be carried out overnight at −20°C so that the samples can be spun down the next morning and the experiment can be continued.

If there are issues with obtaining sufficient yields in the purification of wild‐type and mutant cofilin, buffer composition and wash protocols may be optimized and adjusted as needed.

**Basic Protocol** **5** **—**BCN‐E‐BCN is slightly cytotoxic and may result in cell death if the treatment dose is too high or the incubation time is too long. If labeling other types of live cells, doses and incubation times should be optimized to maximize protein lysate yields. Before treatment, ensure that cells are highly confluent. Another useful step is to exercise caution when washing the cells before lysing.

As was also the case for Basic Protocol 4, the incubation of samples with acetone can be extended from 1 hr at −80°C to overnight at −20°C.

**Basic Protocol** **6** **—**To ensure clean and unobstructed transfer of proteins to nitrocellulose membranes during western blotting, extra care must be taken to roll out any air bubbles during the formation of the transfer sandwich. Skim milk cannot be used for blocking or antibody solutions, as it contains biotin and can greatly interfere with the streptavidin signal during imaging. Therefore, 3% BSA is the optimal choice.

### Understanding Results

**Figure 3:**
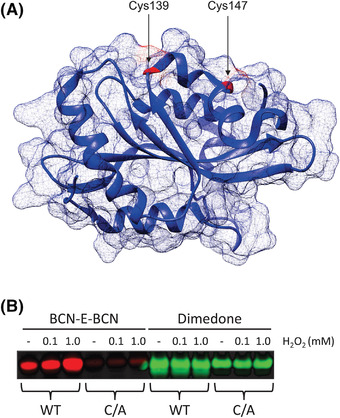
BCN‐E‐BCN labeling of oxidized cofilin compared to dimedone labeling. (**A**) The crystal structure of human cofilin 1 (PDB 4BEX) has been modeled with the two solvent‐exposed cysteine residues Cys139 and Cys147 indicated in red. (**B**) The purified wild‐type cofilin (WT) and C139A/C147A mutant (C/A) were exposed to water (1), 0.1 or 1 mM H_2_O_2_, and then labeled with either BCN‐E‐BCN or dimedone (DMD) and western blotted. BCN‐E‐BCN‐labeled oxidized WT cofilin, with the signal intensity paralleling the H_2_O_2_ concentration, and with significantly less labeling for the C/A mutant. DMD was not able to display nuances in oxidation levels and still strongly labeled the C/A mutant form of cofilin.

Following the protocols listed above, BCN‐E‐BCN can be synthesized from widely available materials, and it can then be used in a variety of labeling applications, including *in vitro* and cell‐based labeling. Following Basic Protocol 4 and the western blotting procedure outlined in Basic Protocol 6, the western blot in Figure 3 was produced. When compared to the *in vitro* labeling capacity of dimedone, it is clear that BCN‐E‐BCN is superior at reflecting nuanced levels of oxidation with increasing H_2_O_2_ concentrations. Although there was some labeling of the wild‐type cofilin sample without added H_2_O_2_, there were marked increases in signal intensity when H_2_O_2_ was added. Consistent with previous results that identified the solvent‐exposed Cys139 and Cys147 residues as the major sites of oxidation, the BCN‐E‐BCN labeling of the C139A/C147A mutant cofilin was considerably lower than that of the wild‐type protein, and it did not increase following incubation with H_2_O_2_. The BCN‐E‐BCN signal in the absence of H_2_O_2_ was likely due to spontaneous oxidation of the protein sample. In contrast, the dimedone labeling remained consistent across all the cofilin samples and conditions, with the C139A/C147A samples being labeled at a similar intensity to the wild‐type sample.

**Figure 4:**
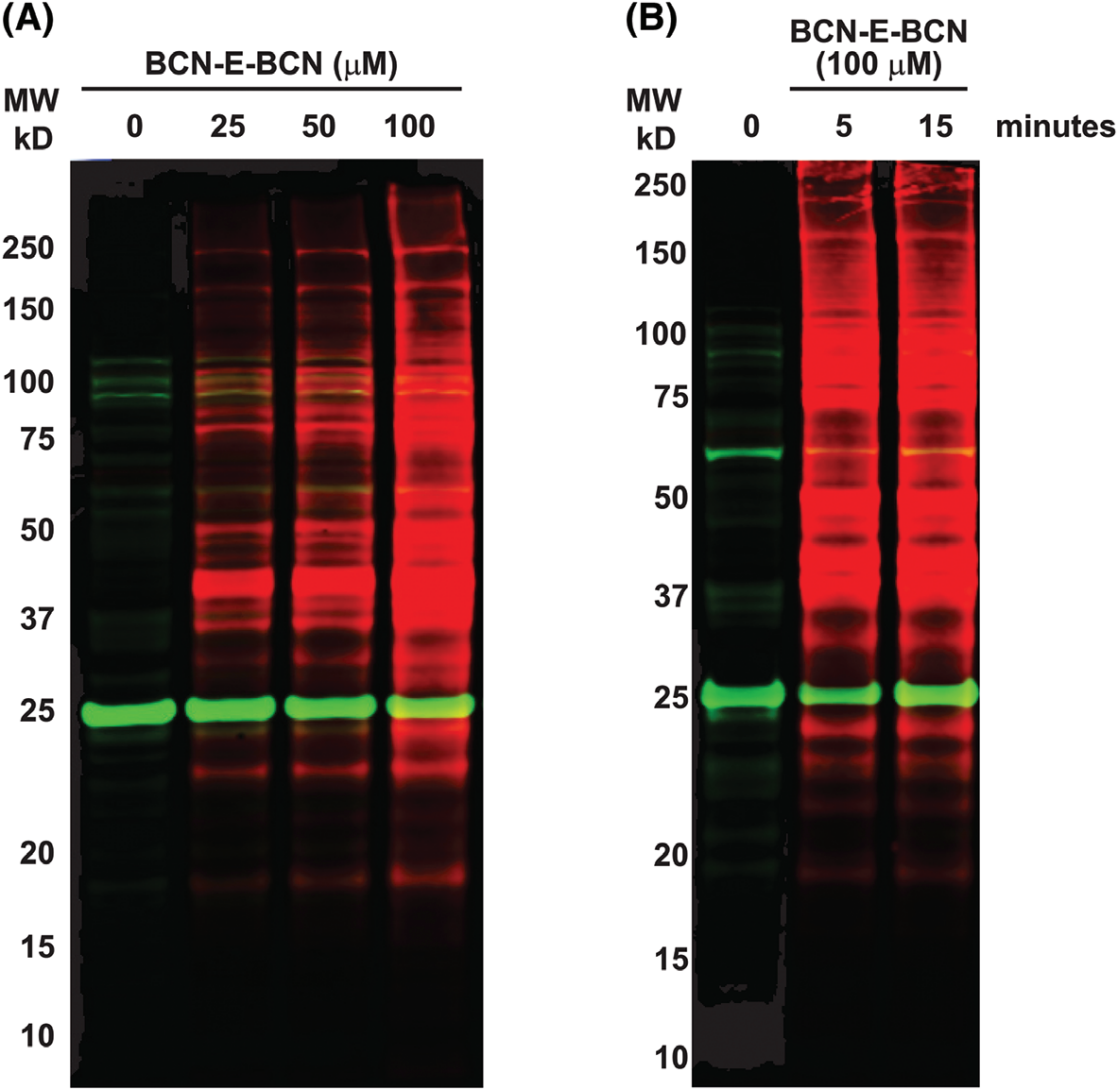
BCN‐E‐BCN labeling of whole cell lysates. (**A**) HEK 293 cells were treated with DMSO vehicle (0) or increasing concentrations of BCN‐E‐BCN for 15 min and were subsequently western blotted after reaction with azide‐PEG3‐biotin. The molecular weights (MWs) of markers in the protein ladder are indicated on the left side of the image. (**B**) HEK 293 cells were treated with DMSO vehicle control (0) or 100 µM BCN‐E‐BCN for 5 or 15 min and subsequently western blotted after reaction with azide‐PEG3‐biotin. Protein MW is indicated on the left side of the image.

With relatively short incubation periods, BCN‐E‐BCN is able to penetrate live cells and label oxidized cysteine residues. Following Basic Protocols 5 and 6 produced the western blot in Figure 4, showing BCN‐E‐BCN labeling of HEK 293 whole cell lysates. The cells shown in Figure 4A were treated with no BCN‐E‐BCN (DMSO vehicle control; or 25, 50, or 100 µM BCN‐E‐BCN for 15 min). All samples underwent parallel azide‐PEG3‐biotin incubation and acetone precipitation. The BCN‐E‐BCN‐treated samples clearly display strong labeling with streptavidin in a dose‐dependent manner, indicating that BCN‐E‐BCN was able to react with a wide variety of proteins within the cell lysate. According to Figure 4B, 100 µM BCN‐E‐BCN was incubated with HEK 293 cells for either 5 or 15 min. Both labeling times yielded comparable protein levels, consistent with the rapid cell penetration and reaction with cysteine sulfenic acids on a range of proteins. There were some background/non‐specific bands present in the sample that did not receive BCN‐E‐BCN treatments, which may be due to certain proteins reacting with the azide‐PEG3‐biotin or binding to fluorescently labeled streptavidin.

### Time Considerations

Basic Protocol 1: The flame‐drying of glassware and drying of copper sulfate catalyst require about 30 min. Preparing for the reaction takes about 1 hr, and the cyclopropanation reaction itself takes 2.5 hr. Workup and purification take approximately half a day. As the two isomers have similar mobility on silica gel, column chromatography alone can require 2 to 4 hr.

Basic Protocol 2: Reduction and bromination can each be consistently completed within 2 hr. Additional time (e.g., up to 1 day) may be needed if refrigeration is required during the recrystallization step. Preparation for the elimination reaction can take 30 to 60 min, whereas the reaction itself needs 2 hr. Workup and purification can take approximately half a day, including rotary evaporation of the reaction solvent (∼30 min), column chromatography (1 to 2 hr), and evaporation of column fractions (∼2 hr). If further purification is needed, recrystallization from hexane or heptane may take up to 1 day of refrigeration.

Basic Protocol 3: Setting up the reaction of **6** with *p*‐nitrophenyl chloroformate takes about 30 min, and the actual reaction is complete within an hour. Workup and purification take 2 to 3 hr, including extractive workup (∼30 min), column chromatography (1 to 2 hr), and evaporation of column fractions (∼1 hr). Likewise, setting up the reaction of **7** with ethylenediamine also takes about 30 min, and the reaction is complete within an hour. The evaporation of *N,N*‐dimethylformamide takes about an hour, but this can vary depending on the condition of the evaporator and strength of vacuum. Column chromatography takes 1 to 2 hr, followed by evaporation of column fractions (∼1 hr).

Basic Protocol 4: Setting up the protein samples, labeling with BCN‐E‐BCN and azide‐PEG3‐biotin, and finally preparing the samples for western blot analysis takes approximately 3 hr. Support Protocol 1, detailing the purification of wild‐type and mutant cofilin, requires a larger time commitment of approximately 4 to 5 days. The transformation of *E. coli* with cofilin expression plasmids takes less than an hour; however, plates need to grow overnight at 37°C. The following afternoon, an overnight culture can be made from the resulting colonies. Larger cultures are grown the next day until the appropriate OD_600_ has been reached; they are then induced and left to grow for an additional 3 hr. The culture is then spun down and pelleted, after which the cell pellet can be frozen and purification can proceed the next day. The preparation and protein purification should take approximately 4 hr.

Basic Protocol 5: The first day of the protocol is dedicated to seeding equal numbers of HEK 293 cells and allowing them to grow to 80%‐90% confluency, which takes 1 to 2 days depending on the starting seeding density. The next day is dedicated to BCN‐E‐BCN treatment, followed by cell lysis, labeling of lysates with azide‐PEG3‐biotin, and preparation of samples for western blotting. Similar to the timeline in Basic Protocol 4, this process, including the lysis procedure, takes approximately 4 hr.

Basic Protocol 6: Depending on whether one is blocking or incubating membranes with primary antibodies overnight at 4°C, the western blotting protocol can take 1 or 2 days. Typically, the gel is run for 1 to 2 hr depending on voltage, and the transfer takes 75 min. If there are time constraints, the protocol can be shortened to 1 day by performing both blocking and primary antibody incubation (if applicable) at room temperature for 1 hr each. Otherwise, either step can be carried out at 4°C overnight. Incubation with streptavidin solution takes 1 hr, followed by approximately 30 min of washing prior to imaging.

### Author Contributions

**Katarina Micovic**: conceptualization, data curation, formal analysis, investigation, methodology, validation, visualization, writing original draft; **Thershan Satkunarajah**: conceptualization, data curation, formal analysis, investigation, methodology, visualization, writing original draft; **Alexandre Carnet**: conceptualization, data curation, formal analysis, investigation, methodology, validation, visualization; **Mackenzie Hurst**: data curation, formal analysis, investigation, methodology, visualization; **Russell Viirre**: conceptualization, formal analysis, methodology, project administration, resources, supervision, writing review & editing; **Michael F. Olson**: conceptualization, data curation, formal analysis, funding acquisition, project administration, supervision, visualization, writing original draft, writing review & editing.

### Conflict of Interest

The authors declare no conflict of interest.

## Supporting information

Supporting Information

## Data Availability

The data, tools, and materials (or their sources) that support the protocol are available from the corresponding author upon reasonable request.
