# Supplementary material for: Synthesis and Use of the Bifunctional Sulfenic Acid Probe BCN‐E‐BCN for In Vitro and Cell‐Based Assays of Protein Oxidation
Source: Curr Protoc. 2022 Oct 6;2(10):e559. doi: 10.1002/cpz1.559 (PMC11648817; doi:10.1002/cpz1.559)
Supplement: Supplementary file 1 — Supporting Information [file CPZ1-2-0-s001.docx]

**Figure 1:** ^1^H NMR Spectrum of 3a (CDCl_3_)

**Figure 2:** ^13^C NMR Spectrum of 3a (CDCl_3_)

**Figure 3:** ^1^H NMR Spectrum of 3b (CDCl_3_)

**Figure 4:** ^13^C NMR Spectrum of 3b (CDCl_3_)

**Figure 5:** ^1^H NMR Spectrum of 4a (CDCl_3_)

**Figure 6:** ^1^H NMR Spectrum of 5a (CDCl_3_)

**Figure 7:** ^13^C NMR Spectrum of 5a (CDCl_3_)

**Figure 8:** ^1^H NMR Spectrum of 6a (CDCl_3_)

**Figure 9:** ^13^C NMR Spectrum of 6a (CDCl_3_)

**Figure 10:** ^1^H NMR Spectrum of 7a (CDCl_3_)

**Figure 11:** ^1^H NMR Spectrum of 1 (CDCl_3_)
